# The impact of introducing Unit Practice Councils on nurse engagement, the nurse practice environment and evidence-based practice: A longitudinal observational study

**DOI:** 10.1016/j.ijnsa.2026.100584

**Published:** 2026-06-06

**Authors:** Marth MLF Jans, Hillegonda A Stallinga, Mick AJ Olvers, Jolanda ter Sluysen, Brigitte JM de Brouwer, Evelyn J Finnema, Rien de Vos, Marjolein IMM Heemels

**Affiliations:** a Maastricht University Medical Center, Department of Nursing, Maastricht, the Netherlands; b University of Groningen, University Medical Center Groningen, Department of Health Sciences, section of Nursing Science, Groningen, the Netherlands; c University Medical Center Nijmegen, Patient Care Institute, Nijmegen, the Netherlands; d Accuralis, Healthcare Research & Consulting, Best, the Netherlands; e Amsterdam University Medical Center, Center for Evidence-Based Education, Amsterdam, the Netherlands

**Keywords:** Diffusion of innovation, Evidence-based practice, Health facility environment, Longitudinal studies, Nurse engagement, Unit Practice Councils, Shared governance, Nursing

## Abstract

**Background:**

Healthcare systems face increasing complexity alongside persistent nursing shortages. Shared Governance, operationalized through Unit Practice Councils, is a professional practice model designed to strengthen nurse engagement, accountability and evidence‑informed decision-making at the point of care. While Shared Governance has been associated with positive nurse outcomes, evidence on the impact of Unit Practice Councils in European academic settings remains limited.

**Objective:**

To evaluate the impact of introducing Unit Practice Councils on nurse engagement, the nurse practice environment and evidence-based practice (EBP) implementation, and to assess the extent to which Unit Practice Councils are embedded into routine practice inpatient units.

**Design:**

Longitudinal observational study.

**Methods:**

Study was conducted across four inpatient units in a Dutch academic hospital. Data were collected at baseline (T0) and at 12-month follow-up (T1) using the Autonomy to Engagement Scan (A tot Z scan), the Practice Environment Scale of Nursing Work Index (PES-NWI) and Evidence Based Practice Implementation Scale (EBP-I). Generalized Estimating Equations were used to analyze changes over time, accounting for interactions between time, council membership, and measurement (paired vs. unpaired). At T1, the Normalization Measure Development (NoMAD) questionnaire was administered to assess the normalization of Unit Practice Councils into routine nursing practice. Differences between council members and non-members were analyzed using the Mann–Whitney U test.

**Study registration:**

Not registered.

**Results:**

A total of 59 nursing staff participated at T0 and 63 at T1, with 36 completing both measurements. Nurse engagement and the nurse practice environment showed small but significant improvements over time (*p* = 0.006, *β*=+0.050 and *p* = 0.048, *β*=+0.026) with significant interaction effects for council membership (*p* = 0.016, *β*=+0.190 and *p* = 0.008, *β*=+0.307), indicating increasing scores among council members. EBP implementation remained unchanged in the full sample (*p* = 0.957). However, council members participating at both time points demonstrated a large and clinically meaningful increase in EBP-I scores (three-way interaction: *p* < 0.001; *β*=+21.98). NoMAD results indicated high normalization levels (median=4.26, IPR=3.86–4.62), with significantly higher scores among council members (*p* = 0.002, *r* = 0.39), indicating that Unit Practice Councils were becoming embedded in routine nursing practice.

**Conclusion:**

Introducing Unit Practice Councils, particularly alongside structured EBP training, resulted in clinically meaningful improvements in EBP among council members with continuing membership. Council members also showed early positive trends in nurse engagement and the nurse practice environment, while outcomes in the broader nursing workforce remained stable over the 12‑month period. High normalization scores indicate successful embedding of Unit Practice Councils within routine practice in an academic hospital.

**What is already known**

• Shared Governance facilitating structures such as Unit Practice Councils are associated with improved nurse outcomes, including higher structural empowerment, increased engagement and a more supportive practice environment.
• Evidenced based practice is common ground for all clinicians, and is fundamental to Shared Governance. Although nursing staff value evidence-based practice (EBP), its implementation is often limited by time constraints, knowledge gaps, and insufficient organizational support.

Alt-text: Unlabelled box dummy alt text

**What this paper adds**

• The introduction of Unit Practice Councils is associated with a clinically meaningful improvement in EBP among continuously participating council members.
• Council members showed early positive trends in perceived nurse engagement and perceptions of the nurse practice environment.
• Unit Practice Councils can be effectively implemented and embedded into routine nursing practice within 24 months, supporting the feasibility of long-term Shared Governance structures.

Alt-text: Unlabelled box dummy alt text

## 1 Introduction

Healthcare systems worldwide are under growing pressure due to demographic change, increasing care complexity, complexity in care organization and persistent workforce shortages (World Health Organization, 2025). Nursing staff provide most of direct patient care and are often the first to detect clinical deterioration (Aiken et al., 2018). High‑quality nursing care is strongly associated with better patient outcomes, including lower in‑hospital mortality and fewer adverse events, underscoring the essential contribution of nursing staff to patient safety and the quality of care (Griffiths et al., 2019).

Despite this responsibility, nursing staff often have limited authority to formally and structurally influence decisions that affect care delivery and outcomes, creating a gap between responsibility and decision‑making authority (Koteswaramma, 2024). High turnover and increasing work pressure further exacerbate these challenges, with potential consequences including reduced job satisfaction, higher attrition and risks to patient safety (Bae, 2024; Mauricio et al., 2025). These pressures highlight the need for structural strategies that strengthen professional nursing practice, enhance their decision-making authority and sustained nurse engagement, thereby enabling trusted decision making at the point of care in increasingly complex environments (Chiou, 2025).

Shared Governance can be understood as a governance philosophy a professional practice model that strengthens nursing practice by positioning authority, accountability and decision-making at the point of care (Allen-Gilliam et al., 2016; Porter-O'Grady, 2017; Porter-OʼGrady, 2019). It emphasizes flat organizational structures, nurse‑led decision‑making and shared accountability for care outcomes (Porter-O'Grady and Finnigan, 1984; O'Grady and Clavelle, 2021). Grounded in principles of partnership, equity, ownership and accountability, Shared Governance aimed to address increasing care complexity and the persistent tension between professional responsibility and hierarchical control, while providing an organizational foundation for sustained nurse engagement (Porter-O'Grady, 2017).

Within this framework, nursing councils function as facilitating structural mechanisms through which Shared Governance is operationalized. Depending on organizational context and design, councils may be established at unit, departmental or organizational levels to support nurse-led decision-making, resource allocation and improvement priorities (Hess, 1998; Hess, 2017; Medeiros, 2018).

A frequently adopted structure within Shared Governance for nurse engagement is the Unit Practice Council.

Unit Practice Councils can be understood as unit-level, nurse-led councils in which frontline staff collectively participate in decision-making regarding clinical practice, quality improvement and the work environment. As such, Unit Practice Councils represent an operationalization of Shared Governance at the point of care, enabling nurses to influence practice through structured, collective decision-making processes (Hess, 2017; O’Grady and Clavelle, 2021). In practice, Unit Practice Councils typically involve reviewing performance data, identifying improvement priorities and implementing changes related to patient care and professional standards (Fray, 2011; Lockhart, 2021). However, the exact operationalization and measurement of Unit Practice Councils functioning varies across studies, reflecting the broader complexity of evaluating Shared Governance structures (Hess et al., 2020).

Evidence suggests that healthcare organizations with Shared Governance structures demonstrate improved patient and nurse outcomes (Kutney-Lee et al., 2015; Rodríguez-García et al., 2020; Janes et al., 2021; Dempsey and Assi, 2018; Van Bogaert et al., 2014). Previous studies have linked Shared Governance to greater structural empowerment, higher nurse engagement and improvements in the professional practice environment, while Unit Practice Councils have been associated with facilitating evidence-based decision-making in nursing practice (Al-Ruzzieh et al., 2022; Olender et al., 2020; Gallagher-Ford, 2015). However, evidence on the early effects of Shared Governance structures in European contexts, particularly within Dutch academic hospitals, remains limited (Kyytsönen et al., 2020).

This gap is particularly relevant in healthcare systems where professional nursing autonomy and formalized shared decision-making structures are less embedded in organizational governance, potentially influencing the implementation and early effects of Shared Governance initiatives. Moreover, the effectiveness of Unit Practice Councils, as structures facilitating Shared Governance, is likely influenced by how they function in practice, including levels of nurse participation, decision-making authority and the implementation of evidence-based practice (EBP). Nurse engagement, the nurse practice environment and EBP implementation can be considered early indicators of Unit Practice Councils effectiveness, reflecting the extent to which Shared Governance principles are embedded in everyday nursing practice.

Assessing these outcomes therefore provides a conceptually coherent and practice-relevant approach to evaluating whether Unit Practice Councils achieve their intended impact.

This study aims to evaluate the impact of introducing Unit Practice Councils^1^ on nurse engagement, the nurse practice environment, and the implementation of EBP, as well as to assess the extent to which the Unit Practice Councils are integrated into routine practice within hospital units.

## 2 Methods

### 2.1 Study design

This longitudinal observational study investigates changes in nurse engagement, the nurse practice environment, and the implementation of EBP during the introduction of Unit Practice Councils over a 12-month period, using two measurement points (baseline and 12-month follow-up).

### 2.2 Setting

At Maastricht University Medical Centre+ (MUMC+) in the Netherlands, an organization wide nursing council structure was implemented to strengthen nursing autonomy, leadership and professional ownership. The structure consists of Unit Practice Councils at ward level, supported by Center and Hospital Councils at *meso* and macro level. Unit Practice Councils function as frontline decision making bodies responsible for improving care quality, strengthening the practice environment and integrating patient perspectives. Councils meet bi weekly and consist of 5–10 members of the nursing staff per unit, representing approximately 25–33% of the nursing staff, with membership proportional to unit size. Unit Practice Councils initiate, implement and monitor practice improvements while serving as a platform for knowledge exchange, professional development and evidence-informed decision-making across the nursing team. Phased implementation of the nursing council structure began in March 2023 with the introduction of four Unit Practice Councils, followed by the establishment of a Center Council in September 2023 and a Hospital Council in September 2024. To support Unit Practice Council functioning a nine-month EBP guidance program (April 2024–January 2025), was provided to council members by Zuyd University of Applied Sciences (the Netherlands). The program included structured guidance, interactive learning sessions, journal clubs, and consultative EBP support, all aimed at strengthen evidence-informed decision-making in Unit Practice Council activities.

This study was conducted at the Maastricht Comprehensive Cancer Center (MCCC) within the MUMC+, the first center within the hospital where Unit Practice Councils were implemented. The MCCC comprises four inpatient nursing units: two surgical units, one medical oncology/hematology unit and one mixed-specialty unit. Together, these units accommodate approximately 6000 annual admissions and employ around 85 full-time equivalent (FTE) nursing staff (Personnel & Organization Department, MUMC+, internal data, 2024).

### 2.3 Sample and data collection

All nursing staff employed on the participating units were eligible for inclusion. For readability, the term ‘nursing staff’ is used throughout this study to refer to all professionals working in nursing roles, including nursing assistants (Individual Healthcare level), registered nurses (vocationally or bachelor-educated), specialized nurses, nurse coordinators, and combined specialized nurse coordinator roles. “Nursing staff’ is used where referring to the overall group, and distinctions are made where relevant. Exclusion criteria included nursing students and nursing staff employed via external staffing agencies or hospital float pools. Staff lists containing institutional email addresses were provided by unit management. All eligible participants received an information letter via institutional email and informed consent was obtained prior to questionnaire completion.

Data were collected at two time points aligned with the implementation timeline: baseline (T0) from March 27 to April 30, 2024, representing the early implementation phase when Unit Practice Council structures were established but substantive functioning was still developing and follow-up (T1) from April 1 to April 25, 2025, representing the post-implementation phase, when the Unit Practice Councils were supposed to be fully embedded and council members^2^ had completed EBP training.

At both time points, participants were invited to complete an online questionnaire administered via the Castor Electronic Data Capture (EDC) system. Invitation emails were followed up to three reminders during each data collection period. Data collection was coordinated by the principal investigator in collaboration with head nurses, team leaders and council chairs. The study was approved by the Medical Ethics Review Committee of the MUMC+ / Maastricht University (2023–0130). All data were pseudonymized and stored securely.

### 2.4 Variables

#### 2.4.1 Nurse engagement

Nurse engagement is commonly defined as a positive and fulfilling work-related state, characterized by vigor, dedication and absorption, as well as meaningful participation in organizational processes (Brandis et al., 2017; García-Sierra and Fernández-Castro, 2018; García-Sierra et al., 2016; Prybil, 2016; Rivera et al., 2011). Nurse engagement was assessed using the *Autonomy to Engagement Scan* (A tot Z Scan), a Dutch instrument designed to measure clinical autonomy and professional governance within healthcare organizations (Accuralis, 2022). Although the instrument is widely used in Dutch Healthcare practice, no peer-reviewed publication is currently available reporting its psychometric properties. Therefore, internal consistency was assessed within the present study.

This study includes three of the eight subscales: 1*.Clinical Autonomy* (10 primary items), *2. Quality Improvement* (7 primary items), and *3. Governance Structures* (9 primary and 2 secondary items), as these domains were considered most likely influenced by the implementation of Unit Practice Councils. Items were rated on a four-point Likert *scale (1= strongly disagree to 4= strongly agree)*.

Internal consistency (Cronbach’s α) was assessed at T0 for each subscale using all available primary and secondary items belonging to that subscale. Cronbach’s α coefficients indicated acceptable internal consistency, with values of 0.711 for *1. Clinical Autonomy*, 0.686 for *2. Quality Improvement*, and 0.716 for *3. Governance Structures*. Overall mean score was calculated and treated as continuous variable, with higher scores indicated greater perceived nurse engagement and autonomy. Although no formal cut-off values are available, a score of 2.5 was used as a neutral reference point, with scores below this value indicating relatively lower levels of engagement and autonomy and scores above indicating relatively higher levels.

#### 2.4.2 The nurse practice environment

The nurse practice environment is defined as factors that enhance or attenuate a nurse’s ability to practice nursing skillfully and deliver high quality care (Lake, 2002). It was measured using the revised Dutch version of the Practice Environment Scale of the Nursing Work Index (PES-NWI), as implemented in the International Nurse forecasting in Europe (RN4CAST) project (Lake, 2002; Sermeus et al., 2011). The instrument consists of 32 items rated on a four-point Likert scale (*1= strongly disagree to 4= strongly agree*), distributed across five subscales: *1. Staffing and Resource Adequacy* (4 items), 2. *Collegial Nurse–Physician Relations* (7 items), *3. Nurse Manager Ability, Leadership and Support* (4 items), *4. Nursing Foundations for Quality of Care* (9 items), and *5. Nurse Participation in Hospital Affairs* (8 items).

Internal consistency of the subscales ranges from Cronbach’s α = 0.71 to 0.84.

Overall mean score was calculated and treated as continuous variable, with higher scores indicating a more favorable nurse practice environment. Scores above 2.5 indicated a favorable environment, while scores below 2.5 were considered unfavorable. Following the enhanced scoring method by Lake and Friese (2006) environments were categorized as: favorable (>2.5 on all or four out of five subscales), mixed (>2.5 on two or three out of five subscales) and unfavorable (>2.5 on one or none of the subscales).

#### 2.4.3 EBP implementation

EBP is defined as an approach to problem-solving in clinical decision-making which integrates best evidence from robust studies with clinicians expertise, and patients’ values and preferences (Melnyk et al., 2012). EBP implementation was assessed using the *Evidence-Based Practice Implementation Scale* (EBP-I) Melnyk et al. (2008), a validated instrument measuring the frequency of EBP-related activities over the past eight weeks (e.g. formulating Population –Intervention- Comparison-Outcome questions, applying evidence in clinical decisions, sharing outcome data with colleagues). For this study, the EBP-I is translated into Dutch with the permission of the original authors (not published; available from author) (Melnyk et al., 2008). The EBP-I includes 18 items rated on a five-point Likert scale *(0= “0 times” to 4= “more than 8 times”)*. The internal consistency is high (Cronbach’s α= 0.95). A total score (0 to 72) were calculated by summing all 18 item scores. Scores were treated as continuous variables with higher values reflecting more frequent engagement in EBP activities.

#### 2.4.4 Implementation of the unit practice council

Implementation processes of the Unit Practice Council were evaluated using the *Normalization MeAsure Development* (NoMAD) questionnaire (Finch et al., 2018), grounded in Normalization Process Theory (NPT) (May and Finch, 2009). The Dutch version used in this study is a translation licensed by the ImpleMentAll consortium since 2018 under a Creative Commons Attribution-NonCommercial 4.0 International License (ImpleMentAll Consortium, 2018). The NoMAD assesses the extent to which complex interventions are normalized in routine practice, which in practical terms reflects the extent to which Unit Practice Councils are embedded in daily nursing work as perceived by professionals directly involved in implementation.

Unlike the other instruments (A-Z scan, PES-NWI, EBP-I), the NoMAD is administered only at T1, reflecting its role as a process evaluation measure. Its results aid in interpreting changes in other outcomes as potentially attributable to Unit Practice Councils functioning. The NoMAD includes 20 items rated on a five-point Likert scale (*1= strongly disagree to 5= strongly agree)*, distributed across four subscales, that represent four generative processes associated with normalization: *1.Coherence* (4 items), *2. Cognitive Participation* (4 items), *3. Collective Action* (7 items), and *4. Reflexive Monitoring* (5 items).

Internal consistency of the subscales ranges from Cronbach’s α= 0.65 to 0.81. Mean subscale and total scores were calculated and treated as continuous variables. Higher scores indicate greater normalization of the Unit Practice Council, i.e., that they are becoming embedded in routine nursing practice.

### 2.5 Study size

A formal sample size calculation was not performed as the study aimed to include all eligible nursing staff on the participating units during the data collection periods.

### 2.6 Statistical methods

#### 2.6.1 Basic characteristics

To examine differences between respondents at T0 and T1 in basic characteristics chi-square tests were conducted, including: gender, age, educational level, nursing role and years of work experience.

#### 2.6.2 Nurse engagement, the nurse practice environment and EBP implementation

To analyze changes over time in nurse engagement (A-Z scan), the nurse practice environment (PES-NWI) and EBP implementation (EBP-I) between T0 and T1, Generalized Estimating Equations (GEE) were applied. GEE was selected because the dataset included both unpaired single-time point measurements and paired repeated measurements. Respondents participating at both T0 and T1, formed the paired subgroup. Those completing only one time point formed the unpaired subgroup. This method accounts for within-subject correlations in the paired subgroup and allows inclusion of participants with only a single unpaired measurement, thereby maximizing data use. An exchangeable correlation structure was specified, assuming constant correlation between repeated measures within individuals.

Analyses proceeded in two steps. First, a main-effects GEE tested whether scores changed statistically significantly over time (T0–T1). Second, interaction terms were added to assess whether changes differed by: *1. council membership* (council member vs. non-council member), *2. measurement type* (dependent paired repeated vs. independent unpaired single-time-point) and *3. the three-way interaction* between time, council membership and measurement type.

Regression coefficients (*β*) and their 95% confidence intervals (95% CI) were used as effect size measures and were derived directly from the GEE analysis. GEE provides population-averaged estimates for predefined contrasts, each expressed relative to a reference category. The following contrasts were specified: T1 vs. T0 (overall time effect); T1 * council membership vs. T0 * non-membership; T1 * dependent vs. T0 * independent; T1 * council membership * dependent vs. T0 * non-membership * independent.

Coefficients represent absolute differences on the original outcome scale. Confidence intervals were calculated using robust standard errors. Effect sizes were interpreted using thresholds tailored to each outcome measure. For the A-Z scan and PES-NWI, interpretation focused on the absolute value of *β* and the width of the CI relative to the 1–4 bounded scale, following recommendations to avoid standardized metrics when standard deviations are small or unstable (Baguley, 2009). Effect sizes (*β*) for the A-Z scan and PES-NWI were classified as none (95% CI includes zero and/or |*β*| < 0.05), small (0.05 ≤ | *β*| < 0.10), moderate (0.10 ≤ |*β* | < 0.15), and large (*|β*| ≥ 0.15).

In contrast, for the EBP-I, effect sizes were evaluated using adapted standardized guidelines with thresholds scaled to the outcome’s standard deviation to ensure clinically meaningful interpretation within the context of population-averaged estimates (Cohen, 1988).

Scores for each questionnaire and subscale were calculated only if ≥ 75% of items are completed. Missing items were not imputed. GEE accommodates missing data by including all available observations for participants which at least one completed measurement. Loss to follow-up was inherently handled by the GEE and by reporting response rates. No sensitivity analyses were conducted.

#### 2.6.3 Implementation of the unit practice council

To analyze differences between council members and non-members in the embedding of the Unit Practice Council in daily practice the NoMAD questionnaire, administered solely at T1, was analyzed using the non-parametric Mann–Whitney U test due to a positively skewed distribution and a limited sample size (n = 63).

Statistical significance was set at *p* < 0.05. Descriptive statistics are reported as median, interquartile range (25%–75% percentile) and range (minimum–maximum) to account for the positively skewed distribution. Effect sizes were calculated as *r*=Z √N following Rosenthal (1991) procedure, where *r* reflects the strength of the difference between council members. Interpretation followed Cohen’s widely accepted thresholds: small (0.10 ≤ *r* < 0.30), medium (0.30 ≤ *r* < 0.50), and large *(r* ≥ 0.50) (Cohen, 1988).

Missing items were treated as incomplete and excluded from the analysis, consistent with the approach used for the other instruments.

All analyses were performed in IBM SPSS Statistics, version 28 (IBM Corp., Armonk, NY, USA). Two-tailed tests were used with statistical significance set at *p* < 0.05. Descriptive statistics are reported as means (SE) with 95% confidence intervals. Independent *t*-tests were used to compare total scores between council members and non-members at both time points.

## 3 Results

### 3.1 Response

At T0 (baseline, March 2024) a total of 125 nursing staff met the inclusion criteria and at T1 (follow-up, April 2025)124 nursing staff were eligible. During this period, 29 (23,2%) nursing staff left the participating units while 28 (22,6%) new nursing staff joined. At T0, 59 nursing staff (including 1 nursing assistant) completed the questionnaire (response rate 47,2%). Of these, 25 (42,4%) were council members and 34 (57,6%) were non-members. At T1, the response rate slightly increased to 50.8%, with 63 completed questionnaires, again including 25 (39.7%) council members and 38 (60,3%) non-members.

Across both measurement points, at both T0 and T1 36 individuals (29,5%) completed the questionnaire, forming the subgroup with dependent, paired measurements. This subgroup consisted of 16 (44,4%) council members and 20 (55,6%) non-members.

### 3.2 Basic characteristics respondents

Across the examined basic characteristics of the respondents, including: gender, age, educational level, nursing role and work experience chi-square analyses (Table 1) revealed no statistically significant differences between respondents at T0 and T1. Although not presented in the table, no statistically significant differences in characteristics were observed between council members and non-council members at either measurement point, indicating that the groups remained comparable over time and across subsets.

**Table 1:** Characteristics of nursing staff respondents at T0 and T1.

Basic characteristics	T0 (total *N* = 59) *n* (%)	T1 (total *N* = 63) *n* (%)	*p*
Sex			
Female	53 (89.8)	56 (88.9)	.866
Age			
<25	6 (10.2)	11 (17.5)	.410
25–35	31 (52.5)	37 (58.7)	
36–45	8 (13.6)	7 (11.1)	
46–55	9 (15.3)	4 (6.3)	
>55	5 (8.4)	4 (6.3)	
Highest level of education			
Training as nursing assistant (IG level)^a^	1 (1.7)	1 (1.6)	.803
MBO-V^b^	21 (35.6)	23 (36.5)	
Bachelor	37 (62.7)	38 (60.3)	
Master	0	1 (1.6)	
Nursing role			
Nursing assistant (IG level)	1 (1.7)	1(1.6)	.499
Registered nurses (total)	58 (98.3)	62 (98.4)	
General ward nurse	39 (66.1)	37 (58.7)	
Specialized nurse	13 (22)	11 (17.5)	
Nurse coordinator	3 (5.1)	8 (12.7)	
Combined specialized nurse coordinator roles	3 (5.1)	6 (9.5)	
Membership in the Unit Practice Council			
Council member: No	34 (57.6)	38 (60.3)	.763
Council member: Yes	25 (42.4)	25 (39.7)	
Years of experience as a registered nurse			
<5	19 (32.2)	19 (30.2)	.331
5–15	25 (42.4)	35 (55.6)	
16–25	7 (11.9)	3 (4.8)	
>25	8 (13.6)	6 (9.5)	
Years of experience as a registered nurse in MCCC			
<5	22 (37.3)	27 (42.9)	.212
5–15	23 (39)	30 (47.6)	
16–25	7 (11.9)	3 (4.8)	
>25	7 (11.9)	3 (4.8)	

a IG level = Individual Healthcare level (nursing assistant level in the Dutch vocational education system).

b MBO-V = intermediate vocational nursing education, including in-service trained nurses. Bachelor = bachelor degree in nursing; Master = master degree in nursing. Registered nurses include vocationally (MBO-V, including in-service trained nurses) and bachelor-educated nurses. Nursing assistants (IG level) are included in nursing staff but are not registered nurses.

At both measurement points, the majority of respondents were female (T0:89.8%; T1:88.9%). Most were aged 25 to 35 years (T0:52.5%; T1:58.7%) and the predominant educational level was a bachelor’s degree (T0:62.7%; T1:60.3%) (Table 1). Most respondents were employed as general ward nurses (T0:66.1%; T1:58.7%), followed by specialized nurses, nurse coordinators, and combined specialized nurse coordinator roles. Regarding work experience, most respondents reported between 0 and 15 years of employment (T0:74,6; T1:85.6%).

### 3.3 Nursing outcomes

#### 3.3.1 Nurse engagement

The extent to which the introduction of Unit Practice Councils influenced nurse engagement, as measured by the mean total score of three subscales of the A–Z Scan (Table 2), showed a statistically significant positive main effect of time (*p* = 0.006). The mean score increased slightly from 2.67 at T0 to 2.78 at T1. Although the estimated effect was classified as none, (*β*=+0.050, 95% CI –0.105 to +0.206), the positive *β* suggests a possible trend toward improved engagement over time.

**Table 2:** Impact of the implementation of the Unit Practice Council on nursing practice: engagement, practice environment and evidence-based practice (total scores).

Indicator	Analysis	Wald χ²	*p*		T0 mean (SE) {95% CI}	T1 mean (SE) {95% CI}	*β* (SE) {95% CI}
Engagement							
A-Z scan^a^	Measurement point^1^	7.514	.006		2.67 (0.03) {2.60–2.72}	2.78 (0.04) {2.71–2.86}	+0.050 (0.079) {–0.105 – +0.206}
	Measurement point *council membership	5.780	.016	council: no	2.72 (0.04) {2.64–2.79}	2.73 (0.04) {2.64–2.81}	
				council: yes	2.61 (0.05) {2.52–2.71}	2.84 (0.06) {2.72–2.95}	+0.190 (0.151) {–0.106 – +0.486}
	Measurement point * measurement type^2^	0.440	.507	independent	2.65 (0.05) {2.56–2.75}	2.80 (0.06) {2.68–2.91}	
				dependent	2.68 (0.04) {2.60–2.75}	2.77 (0.05) {2.68–2.86}	–0.078 (0.099) {–0.273 – +0.116}
	Measurement point *council membership *measurement type	0.208	.901	council:(no) - independent	2.69 (0.06) {2.57–2.81}	2.74 (0.06) {2.62–2.86}	
				council:(no) - dependent	2.74 (0.04) {2.66–2.82}	2.71 (0.06) {2.60–2.83}	
				council:(yes) - independent	2.61 (0.07) {2.47–2.76}	2.85 (0.10) {2.65 −3.06}	
				council:(yes) - dependent	2.61 (0.06) {2.50–2.73}	2.82 (0.06) {2.70–2.93}	–0.009 (0.144) {–0.291 – +0.272}
Practice environment							
PES-NWI^b^	Measurement point	3.920	.048		2.75 (0.04) {2.68–2.82}	2.85 (0.04) {2.78–2.92}	+0.026 (0.818) {−0.135 – +0.186}
	Measurement point *council membership	7.095	.008	council: no	2.83 (0.04) {2.74–2.92}	2.79 (0.03) {2.73–2.86}	
				council: yes	2.67 (0.05) {2.57–2.78}	2.91 (0.07) {2.78–3.04}	+0.307 (0.182) {−0.051 – +0.664}
	Measurement point *measurement type	2.477	.116	independent	2.72 (0.06) {2.60–2.84}	2.90 (0.07) {2.77–3.04}	
				dependent	2.78 (0.04) {2.71–2.85}	2.80 (0.03) {2.74–2.86}	−0.126 (0.113) {−0.347 - +0.096}
	Measurement point *council membership *measurement type	0.724	.696	council:(no) - independent	2.79 (0.07) {2.66–2.92}	2.81 (0.05) {2.71–2.92}	
				council:(no) - dependent	2.87 (0.06) {2.75–2.98}	2.77 (0.04) {2.69–2.85}	
				council:(yes) - independent	2.66 (0.10) {2.46–2.86}	2.99 (0.13) {2.74–3.24}	
				council:(yes) - dependent	2.69 (0.04) {2.61–2.77}	2.83 (0.05) {2.74–2.92}	−0.117 (0.151) {−0.412-+0.179}
Evidence-based practice							
EBP-I^c^	Measurement point	0.003	.957		14.13 (1.58) {11.03–17.23}	14.25 (2.02) {10.29–18.20}	+0.052 (4.487) {–8.743 – +8.846}
	Measurement point *council membership	0.825	.364	council: no	13.63 (2.30) {9.10–18.15}	11.41 (1.70) {8.09–14.74}	
				council: yes	14.86 (3.15) {8.68–21.05}	16.84 (2.45) {12.03–21.66}	–4.552 (8.142) {–20.509 – +11.406}
	Measurement point *measurement type	0.901	.342	independent	14.00 (3.40) {7.33–20.05}	11.78 (2.35) {7.18–16.38}	
				dependent	14.49 (2.18) {10.22–18.75}	16.47 (2.12) {12.32–20.63}	–4.538 (4.974) {–14.286 – +5.210}
	Measurement point *council membership *measurement type	15.664	<0.001	council:(no) - independent	14.51 (3.71) {7.25–21.77}	14.56 (2.91) {8.86–20.26}	
				council:(no) - dependent	12.75 (2.75) {7.35–18.15}	8.26 (1.74) {4.85–11.86}	
				council:(yes) - independent	13.50 (5.71) {2.31–24.69}	9.00 (3.68) {1.78–16.22}	
				council:(yes) - dependent	16.23 (2.68) {10.98–21.48}	24.68 (3.25) {18.31–31.06}	+21.98 (5.59) {+11.02- +32.94}

β represents the estimated effect size; interpretation should consider both β and the 95% confidence interval. Positive β values indicate an increase from T0 to T1.

p-values indicate statistical significance: p < 0.05.

a A-Z-scan = Autonomy to Engagement Scan - scale range: 1–4 (1 = strongly disagree; 4 = strongly agree).

b PES-NWI = Practice Environment Scale of the Nursing Work Index - scale range: 1–4 (1 = strongly disagree; 4 = strongly agree).

c EBP-I = Evidence Based Practice Implementation Scale - scale range: 0–72 (0–4 frequency scale; 0 = never; 4 = > 8 times).

1 Measurement point: T0=April 2024; T1=April 2025.

2 Measurement type: independent = questionnaire completed once (T0 or T1) – dependent = questionnaire completed at both measurement points (T0 or T1).

Further, total mean scores consistently exceeded the neutral reference threshold of 2.5 at both measurement points (range 1–4), indicating that engagement levels were and remained relatively high.

The observed overall increase was driven by council membership. The interaction between time and council membership was statistically significant (*p* = 0.016), indicating that engagement changed differently over time for council members versus non-council members. Engagement scores among non-council members remained stable (T0:2.72; T1:2.73), whereas council members showed a statistically significant increase (T0:2.61; T1:2.84). The estimated change for council members was +0.190 (SE=0.151), exceeding the threshold for a large effect size (|β| ≥ 0.15). However, as the 95% CI (–0.106 to 0.486) included zero, this estimate should be interpreted with caution.

The positive direction of the *β* alongside the statistically significant interaction suggests a possible trend toward increased engagement among council members.

#### 3.3.2 The nurse practice environment

The extent to which the introduction of Unit Practice Councils influenced the perceived nurse practice environment, assessed using the PES-NWI (Table 2), showed a statistically significant main effect of time (*p* = 0.048), with a slight increase in the mean total score across overall sample (T0:2.75; T1:2.85). The estimated change for overall sample was *β*=+0.026 and the 95% CI (–0.135 to +0.186) indicating a trend toward improved nurse practice environment. Total mean scores remained above the neutral reference value of 2.5 at both time points, indicating a generally favorable environment. This conclusion was further supported by the Lake and Friese (2006) enhanced scoring method, with four out of five subscales exceeding 2.5 (Appendix 1), indicating overall positive perceptions of the practice environment, except for subscale *1. Staffing and Resource Adequacy* which remained below this threshold at both measurement points.

Like nurse engagement, the observed overall improvement was primarily driven by council members. The interaction between time and council membership was statistically significant (*p* = 0.008), indicating differential patterns over time between groups. Non-council members showed a slight decrease (T0:2.83; T1:2.79), whereas council members reported an increase (T0:2.67; T1:2.91). The estimated change for council members was +0.307 (SE=0.182), corresponding to a relatively large effect size, although the 95% CI (–0.051 to +0.664) included zero, the positive direction in the coefficients alongside the significant interaction suggest a possible trend toward a more favorable practice environment among nursing staff participating in the Unit Practice Council.

#### 3.3.3 EBP implementation

The impact of Unit Practice Council implementation on EBP, measured with the EBP-I (Table 2), showed minimal change across the overall sample, with mean scores of 14.13 at T0 and 14.25 at T1. At the overall level, no statistically significant main effect of time (*p* = 0.957) or interaction between time and council membership (*p* = 0.364) was observed. However, a statistically significant three-way interaction among time (T0-T1), council membership and measurement type (independent vs. dependent measurements) was identified (*p* < 0.001*, exact value not available*), indicating that changes in EBP-I scores over time differed depending on both council membership and measurement type. Council members who completed the questionnaire at both time points showed a substantial increase in EBP-I scores (T0:16.23; T1:24.68), with an estimated effect classified as large (*β*=21.98) and clinically meaningful, as the 95% CI excluded zero (+11.02 to +32.94). This interpretation was based on the scale’s standard deviation (SD ≈ 14.69), with effects categorized as none (95% CI includes zero and/or |*β*|<5), small (5 ≤ |*β*|< 7.5), moderate (7.5 ≤ |*β*|<12), and large (|*β*| ≥12). Taken together, these results suggest that EBP implementation remained stable across the full sample, while nursing staff actively participated in the Unit Practice Council and measured longitudinally showed a substantial increase in their performance of EBP activities in daily practice, demonstrating notable and clinically meaningful improvements in EBP.

#### 3.3.4 Implementation of the unit practice council

Assessment of the perceived embedding of Unit Practice Councils 24 months after their introduction, measured with the NoMAD questionnaire is presented in Table 3. Responses were obtained from 63 nursing staff, including 25 council members and 38 non-members. Overall, perceived embedding was high, with a median NoMAD score of 4.26 (IPR:3.86–4.62). Among the four NoMAD subscales, *2. Cognitive Participation* showed the highest ratings (median 4.75, IPR:4.00–5.00), whereas *4. Reflexive Monitoring* showed the lowest (median 4.00, IPR:3.60–4.60).

**Table 3:** Implementation of the Unit Practice Council: Results of the NoMAD questionnaire (total and subscale scores).

NoMAD questionnaire	analysis	Mann- Whitney U	*z*	*p*	*r*	median	percentiles [25–75%]	range [min-max]
NoMAD^a^ questionnaire	total:					4.26	[3.86–4.62]	[1.18–5.00]
	council: no	253.5	−3.112	.002	0.39	4.12	[3.71–4.43]	[1.21–4.95]
	council: yes					4.52	[4.20–4.83]	[1.18–5.00]
1. Coherence	total:					4.25	[3.75–4.75]	[1.00–5.00]
	council: no	419	−0.794	.427	0.10	4.13	[3.50–4.56]	[1.00–5.00]
	council: yes					4.25	[3.87–4.75]	[1.25–5.00]
2. Cognitive Participation	total:					4.75	[4.00–5.00]	[1.00–5.00]
	council: no	208.5	−3.841	<0.001	0.48	4.25	[3.69–4.75]	[1.25–5.00]
	council: yes					5.00	[4.75–5.00]	[1.00–5.00]
3. Collective Action	total:					4.14	[3.71–4.57]	[1.00–5.00]
	council: no	259.5	−3.040	.002	0.38	4.07	[3.57–4.29]	[1.00–5.00]
	council: yes					4.57	[4.00–4.93]	[1.29–5.00]
4. Reflexive Monitoring	total:					4.00	[3.60–4.60]	[1.20–5.00]
	council: no	238.5	−3.345	<0.001	0.42	3.80	[3.40–4.40]	[1.40–4.80]
	council: yes					4.60	[4.00–4.80]	[1.20–5.00]

NoMAD was administered 2 years after the introduction of the councils, corresponding to measurement point T1.

p-values indicate statistical significance: p < 0.05.

r represents the estimated effect size between council members and non-council members.

a NoMAD = Normalization Measure Development questionnaire – scale range: 1–5 (1 = strongly disagree; 5 = strongly agree).

Council members reported statistically significantly higher overall NoMAD scores than non-members (*p* = 0.002).The estimated change for council members was +0.39, which corresponds to a medium effect size (0.3≤*r* < 0.5). This pattern was largely consistent across most subscales. Statistically significant differences were observed for *2. Cognitive Participation* (*p* < 0.001, *exact value not available*)*, 3. Collective Action* (*p* = 0.002)*,* and *4. Reflexive Monitoring* (*p* < 0.001, *exact value not available*), all reflecting medium effect sizes. In contrast, *1.Coherence* showed no statistically significant difference between council members and non-council members (*p* = 0.427, *r* = 0.10), corresponding to a small effect size (0.1≤*r* < 0.3). These findings indicate a generally high level of normalization, suggesting that the Unit Practice Councils are well embedded in routine nursing practice and have been successfully implemented into daily workflows.

## 4 Discussion

### 4.1 Key results

The aim of this study was to establish the impact of introducing Unit Practice Councils on nurse engagement, the nurse practice environment and EBP as well to assess the degree to which the Unit Practice Councils were embedded within hospital units of a university medical center. Nursing staff participating in Unit Practice Councils and followed longitudinally, demonstrated pronounced improvements.

The most notably finding concerned EBP implementation. Council members with continuing membership showed a clear and clinically meaningful increase in EBP activities, while scores in the overall sample remained unchanged. For nurse engagement and the nurse practice environment, council members demonstrated positive trends over time, whereas non‑members scores remained stable. These differences, even within a relatively small group of respondents, suggest a potential trend toward enhanced engagement and a more favorable practice environment among nursing staff participating in Unit Practice Councils.

Implementation findings indicated that Unit Practice Councils were well embedded into routine nursing practice after 24 months, with overall NoMAD scores high across all subscales. Council members rated implementation higher than non-members, suggesting that Unit Practice Councils were firmly integrated into their daily practice.

### 4.2 Interpretations

Overall, the findings suggest that the introduction of nursing councils, as part of the Shared Governance structure, is associated with positive trends in engagement, the practice environment and EBP, particularly among nursing staff actively participating in Unit Practice Councils.

The Unit Practice Council appears to provide a structured environment that fosters professional involvement, autonomy and accountability, while supporting the application of evidence-based knowledge in clinical practice

The most pronounced effect was observed in EBP implementation, with council members continuing participating showing substantial improvements in EBP‑I scores.

This likely reflects the combination of sustained engagement, structured EBP training and governance mechanisms explicitly designed to support the practical application of evidence based knowledge. These results align with literature emphasizing the importance of dedicated EBP education and skill building to acculturate nursing staff to EBP (Friesen et al., 2017; Speroni et al., 2020). While nursing staff generally hold positive beliefs about EBP, persistent barriers, such as lack of time, heavy workload and knowledge deficits, often hinder implementation (Warren et al., 2016). The deliberate combination of targeted EBP training and active participation in Shared Governance structures addresses these barriers by providing both organizational support and the necessary skills (Warren et al., 2016).

In contrast, non-council members and intermittently participating council members did not show comparable improvements, underscoring the importance of continuity for translating EBP knowledge into practice.

Taken together, Unit Practice Councils may serve as an effective mechanism to support practical EBP implementation when combined with continuing participation and structured facilitation. The pronounced improvement in EBP implementation aligns with previous research highlighting the role of Shared Governance in fostering professional development and clinical innovation (Abu Dawass et al., 2023; Fisher and Hubbard, 2015; Gallagher-Ford, 2015).

The study findings suggest that Unit Practice Council participation may enhance nursing staff’s professional autonomy and perceived influence. The council structure provides a formal mechanism for nursing staff to participate in governance, influence policy and contribute to quality initiatives. This is theoretically consistent with prior evidence demonstrating that Shared Governance empowers nursing staff by increasing decision-making authority and professional accountability (Abu Dawass et al., 2023; Allen-Gilliam et al., 2016). Unchanged scores among non-council members indicate that the benefits of Shared Governance and Unit Practice Council activities have not yet difussed across the broader nursing workforce, reflecting a common stage in implementation where increased autonomy and authority remain concentrated among those directly involved (Hamad, 2018). Other studies have similarly reported that engagement gains often emerge first among active participants before diffusing more widely (Ong et al., 2017). Shared Governance requires nursing staff to take ownership and accountability for competence, quality and practice. Unit Practice Council participation provides a platform for demonstrating this responsibility (Lott and Partridge, 2025; O'Grady and Clavelle, 2021).

Taken together, these findings indicate that Unit Practice Council participation provide a mechanism for enhancing professional autonomy and engagement, with potential implications for the diffusion of Shared Governance principles across the wider nursing workforce.

The findings of this study suggest that Unit Practice Council participation contribute to more positive perceptions of the practice environment, creating opportunities for nursing staff to engage in unit-level decision-making and contribute to organizational processes, consistent with their intended role. Higher ratings on *Nursing Foundations for Quality of Care* indicate that Unit Practice Council participation strengthens nursing staff’s professional vision regarding care quality, while improvements in *Nurse Manager Ability, Leadership, and Support* may partly reflect concurrent organizational initiatives, such as leadership development programs emphasizing coaching-oriented management. In contrast, *Staffing and Resource Adequacy* remained below the neutral reference point of 2.5 at both time points, suggesting that structural workforce challenges require broader organizational interventions beyond unit-level governance.

These findings align with previous research showing that council members often rate their practice environment highly and perceive empowerment through Shared Governance structures (Abu Dawass et al., 2023; Al-Ruzzieh et al., 2022). Longitudinal studies have demonstrated consistent progress in professional practice environments during Shared Governance implementation, highlighting the role of Unit Practice Councils in shaping these perceptions (Al-Ruzzieh et al., 2022; Allen-Gilliam et al., 2016).

Overall, these findings suggest that Unit Practice Councils may foster a more supportive and quality-oriented practice environment for nursing staff participating in Unit Practice Councils, while underscoring the need for complementary strategies to extend these benefits across the wider workforce, such as strengthening organisational quality standards, enhancing supportive nurse leadership, expanding meaningful opportunities for professional development, and clarifying and supporting professional nursing standards (Pursio et al., 2024).

High overall NoMAD scores indicate successful implementation of Unit Practice Councils into routine nursing practice 24 months post introduction. Consistently high ratings across subscales reflect recognition of the Unit Practice Councils value and legitimacy, shared understanding of their purpose and collective commitment to success.

Council members showed statistically significantly higher scores on *Cognitive Participation, Collective Action an*d *Reflexive Monitoring*, reflecting deeper operational involvement and engagement. In contrast, *Coherence* scores indicate that most nursing staff, understand the Unit Practice Councils purpose regardless of membership. These findings suggest that active participation drives the operational and experiential aspects of Unit Practice Councils, while a broader understanding of purpose is shared across the workforce, even at this early stage of implementation..

#### 4.2.1 Synthesis

Taken together, these findings indicate that Unit Practice Councils can support EBP, nurse engagement and a more favorable practice environment for participating nursing staff. Positive trends were concentrated among actively participating council members, emphasizing the importance of continuity and structured participation. Broader workforce benefits may require longer implementation periods, targeted diffusion strategies and complementary organizational support. These results align with existing literature on Shared Governance, highlighting the value of structured councils in enhancing professional autonomy, engagement, and the practical application of evidence-based practice (Abu Dawass et al., 2023; Allen-Gilliam et al., 2016; Lott and Partridge, 2025).

### 4.3 Limitations

Several limitations should be considered. First, Unit Practice Councils had been implemented for only 24 months, resulting in approximately one year between baseline and follow-up.

This timeframe captures the early please of Unit Practice Council implementation an may not be sufficient for detecting meaningful changes in complex organizational outcomes such as nurse engagement and practice environment. Second, the overall number of eligible respondents was relatively small. Although response rates were acceptable (T0:47.2%; T1:50.8%), the subgroup completing assessments at both time points was limited (n = 36). Generalized Estimating Equations were used to include both dependent and independent observations maximizing statistical power. Wide confidence intervals for engagement and practice environment, despite large positive *β-*coefficients, likely reflect sample size and heterogeneity rather than absence of effect.

Third, findings should be interpreted within the broader context of Shared Governance. Unit Practice Councils are embedded within wider Shared Governance structures, and Shared Governance was not directly measured as a distinct construct or independent variable. As a result, the observed effects cannot be attributed exclusively to Unit Practice Councils in isolation. However, this reflects real-world clinical practice, in which such organizational structures are inherently intertwined and implemented as part of a broader governance model.

Finally, council members were somewhat overrepresented in the respondent group (approximately 40%) compared with their proportion in the workforce (estimated 25–33%). This may have led to a modest overestimation of perceived improvements, particularly for EBP implementation, but the observed patterns across outcomes are consistent with longitudinal trends and the relative size of the overrepresentation is unlikely to fully account for the magnitude of the effects.

### 4.4 Generalizability

The generalizability of these findings is limited by contextual and methodological factors. This study was conducted within a single Dutch University Medical Center, involving four inpatient units with their own leadership structures, staffing patterns and histories of Shared Governance. Effects, particularly the clinically meaningful improvement in EBP among council members, are closely tied to organizational culture, readiness for change and internal support.

Shared Governance structures vary across hospitals in terms of mandate, decision-making authority, leadership involvement, meeting frequency and integration with organizational processes. Therefore, results may not directly translate to institutions employing different models.

Workforce characteristics also limit generalizability. In our setting, most nursing staff were bachelor-educated and had less than 15 years of experience. Staffing profiles differ considerably between hospitals and variation in educational background and seniority may influence how Unit Practice Councils are implemented and experienced.

Future research could further explore potential differences between professional group.

High NoMAD scores suggest that strong managerial support and resources which may not be present in other settings.

In summary, the study provides strong internal evidence that active Unit Practice Council participation can support improvements, particularly in EBP implementation. The single-center, observational design and contextual factors limit broader generalizability, highlighting the need for multi-center studies to determine the applicability of these findings across diverse hospital contexts and council models.

## 5 Conclusion

Unit Practice Councils can be successfully implemented into routine practice within 24 months, especially when organizational readiness and managerial support are present. Shared Governance structures, as Unit Practice Councils, may strengthen nursing practice when nursing staff are actively and continuously engaged. Council members with continuing participation demonstrated clinically meaningful improvements in EBP and positive trends in engagement and the practice environment, supported by structured facilitation including targeted EBP training and governance processes.

Broader implementation research and larger samples are needed to determine how these effects can be expanded and replicated across diverse healthcare settings.

## Funding

This work was supported by ZonMw [43866].

## Declaration of generative AI and AI-assisted technologies

During the preparation of this work the authors solely used Copilot in order to support minor improvements in grammar and clarity of the manuscript text. After using this tool, the authors reviewed and edited the content as needed and take full responsibility for the content of the publication.

## CRediT authorship contribution statement

**Marth MLF Jans:** Writing – review & editing, Writing – original draft, Visualization, Project administration, Methodology, Investigation, Formal analysis, Data curation, Conceptualization. **Hillegonda A Stallinga:** Writing – review & editing, Writing – original draft, Validation, Supervision, Methodology, Investigation, Formal analysis, Conceptualization. **Mick AJ Olvers:** Writing – review & editing, Validation, Conceptualization. **Jolanda ter Sluysen:** Writing – review & editing. **Brigitte JM de Brouwer:** Writing – review & editing, Resources. **Evelyn J Finnema:** Writing – review & editing, Validation, Supervision, Methodology, Funding acquisition, Conceptualization. **Rien de Vos:** Writing – review & editing, Validation, Supervision, Methodology, Funding acquisition, Conceptualization. **Marjolein IMM Heemels:** Writing – review & editing, Validation, Funding acquisition, Conceptualization.

## Declaration of competing interest

Accuralis is the developer and owner of the A tot Z Scan instrument. Accuralis had no role in the study design, data collection, data analysis or the decision to submit the manuscript. The other authors declare that they have no conflicts of interest.

## Appendix Supplementary materials

Image, application 1

## Footnotes

1 In this study, a Unit Practice Council refers to a nursing-led council.

2 Council members are nurses who actively participate in the Unit Practice Council. Non-council members are nurses who do not participate in the Unit Practice Council.
