# Supplementary material for: The impact of introducing Unit Practice Councils on nurse engagement, the nurse practice environment and evidence-based practice: A longitudinal observational study
Source: Int J Nurs Stud Adv. 2026 Jun 6;11:100584. doi: 10.1016/j.ijnsa.2026.100584 (PMC13276460; doi:10.1016/j.ijnsa.2026.100584)
Supplement: Supplementary file 1 [file mmc1.docx]

**Appendix**

Appendix 1. Subscale scores on the Practice environment: the PES-NWI^1^

| Subscale | T0 mean (SE) {95% CI} | T1 mean (SE) {95% CI} |
| --- | --- | --- |
| 1. Staffing and Resource Adequacy | 2.34 (0.07) {2.21-2.47} | 2.33 (0.08) {2.17-2.48} |
| 2. Collegial Nurse-Physician Relations | 3.10 (0.06) {2.98-3.21} | 3.11 (0.06) {2.99-3.23} |
| 3. Nurse Manager Ability, Leadership, & Support of Nurses | 2.98 (0.05) {2.88-3.07} | 3.16 (0.06) {3.03-3.28} |
| 4. Nursing Foundations for Quality of Care | 2.74 (0.06) {2.62-2.86} | 2.90 (0.04) {2.81-2.98} |
| 5. Nurse Participation in Hospital Affairs | 2.59 (0.04) {2.50-2.68} | 2.70 (0.05) {2.61-2.79} |

*Scale range PES-NWI: 1-4 (1.strongly disagree – 4.strongly agree)*
*^1^the Practice Environment Scale of the Nursing Work Index*
